# Large seasonal and spatial variation in nano- and microphytoplankton diversity along a Baltic Sea—North Sea salinity gradient

**DOI:** 10.1038/s41598-020-74428-8

**Published:** 2020-10-19

**Authors:** Malin Olofsson, James G. Hagan, Bengt Karlson, Lars Gamfeldt

**Affiliations:** 1 Research and Development, Oceanography, Swedish Meteorological and Hydrological Institute, Sven Källfelts gata 15, 426 71 Västra Frölunda, Sweden; 2 Department of Marine Sciences, University of Gothenburg, Box 100, 405 30 Gothenburg, Sweden; 3 Gothenburg Global Biodiversity Centre, Box 461, 405 30 Gothenburg, Sweden; 4 Centre for Sea and Society, Box 260, 405 30 Gothenburg, Sweden; 5 Present Address: Department of Aquatic Sciences and Assessment, Swedish University of Agricultural Sciences, Box 7050, 750 07 Uppsala, Sweden

**Keywords:** Biodiversity, Population dynamics, Marine biology

## Abstract

Aquatic phytoplankton experience large fluctuations in environmental conditions during seasonal succession and across salinity gradients, but the impact of this variation on their diversity is poorly understood. We examined spatio-temporal variation in nano- and microphytoplankton (> 2 µm) community structure using almost two decades of light-microscope based monitoring data. The dataset encompasses 19 stations that span a salinity gradient from 2.8 to 35 along the Swedish coastline. Spatially, both regional and local phytoplankton diversity increased with broad-scale salinity variation. Diatoms dominated at high salinity and the proportion of cyanobacteria increased with decreasing salinity. Temporally, cell abundance peaked in winter-spring at high salinity but in summer at low salinity. This was likely due to large filamentous cyanobacteria blooms that occur in summer in low salinity areas, but which are absent in higher salinities. In contrast, phytoplankton local diversity peaked in spring at low salinity but in fall and winter at high salinity. Whilst differences in seasonal variation in cell abundance were reasonably well-explained by variation in salinity and nutrient availability, variation in local-scale phytoplankton diversity was poorly predicted by environmental variables. Overall, we provide insights into the causes of spatio-temporal variation in coastal phytoplankton community structure while also identifying knowledge gaps.

## Introduction

Phytoplankton are a diverse group of unicellular, photosynthetic micro-organisms. Due to their short generation times, high growth rates and dispersal abilities, phytoplankton are largely cosmopolitan and have colonized almost all photic aquatic environments^1^. As a result, phytoplankton are estimated to be responsible for over 45% of earth’s annual net primary production^2^. Given the ubiquity and productivity of phytoplankton, they are considered key organisms in global ocean nutrient and carbon cycles.

The world’s oceans are currently changing rapidly^3,4^, but our understanding of the consequences for phytoplankton biodiversity and ecosystem functioning are limited. Overexploitation, sea use change, climate change, pollution and eutrophication cause shifts in the distribution of marine organisms^3,5^, and declines in biodiversity generally have negative effects on ecosystem functioning^6,7^. However, biodiversity is not always related to functioning in predictable ways^8^. For example, pelagic ecosystems subject to eutrophication are frequently dominated by just a few phytoplankton taxa (e.g. large algal blooms of filamentous cyanobacteria) which are characterised by high primary productivity and nitrogen fixation^9^. Thus, understanding how phytoplankton biodiversity and abundance relate to variation in the marine environment is important to understand the consequences of ecosystem change.

Phytoplankton communities respond strongly to spatial salinity gradients as species and groups vary considerably in their salinity tolerance^10^. Global phytoplankton diversity is dominated by diatoms and dinoflagellates (in terms of described species)^11^ which are generally well-adapted to high salinity conditions. In contrast, filamentous cyanobacteria are poorly adapted to high salinity conditions but dominate in lower salinity, brackish conditions^12–15^. Differential salinity tolerances among phytoplankton groups with different sized species pools led to the hypothesis that phytoplankton species diversity peaks at high and low salinities (on a gradient from seawater to freshwater)^16^. Although recent analyses support this hypothesis^17,18^, there is still limited exploration of how phytoplankton diversity and community structure vary along wide salinity gradients.

Whilst variation in salinity affects phytoplankton community structure in space, phytoplankton community structure also varies seasonally, particularly in temperate and boreal climate zones. In early spring, phytoplankton grow rapidly due to increasing sunlight and accumulated nutrients, whereas grazing and parasitic attacks limit phytoplankton biomass later in the season^19^. Moreover, high nutrient loads generally increase phytoplankton biomass but tend to decrease species diversity^20^. For example, large summer blooms driven by nutrient availability can be dominated by just a few species of filamentous cyanobacteria. Thus, nutrient availability can shape phytoplankton community composition, which has been shown, for example, in certain regions of the Baltic Sea^21^.

In this study, we explored spatial and temporal variation in phytoplankton community structure (specifically, biodiversity and abundance) across a broad salinity gradient along the Swedish coastline. Specifically, we addressed two questions:

- **Question 1:** Does phytoplankton community structure vary spatially along a broad salinity gradient?
- **Question 2:** How does phytoplankton community structure vary seasonally, does seasonal variation change along the salinity gradient, and which environmental variables explain this variation?

**Figure 1:**
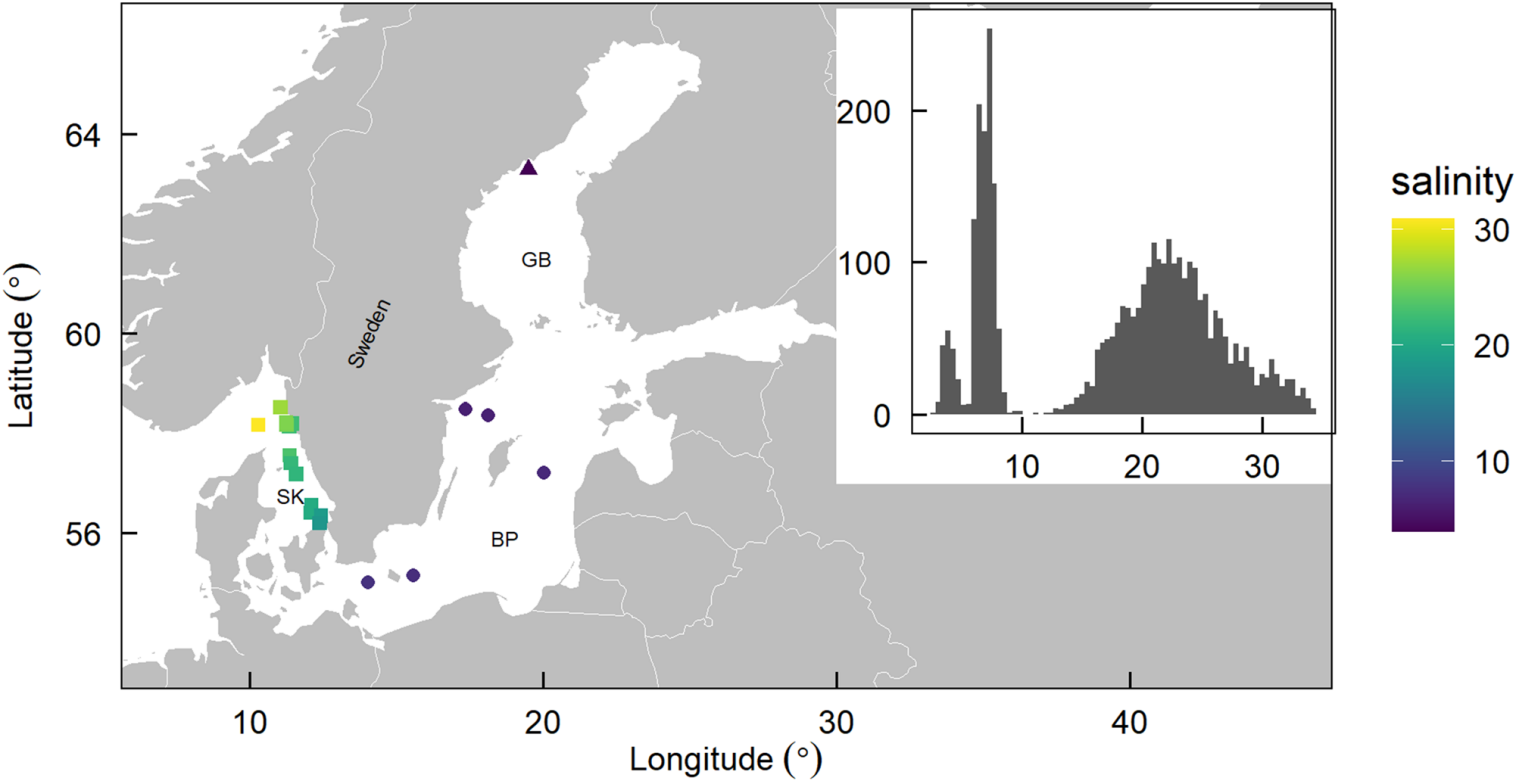
Map of sampling stations and salinity. Map of the 19 sampling stations distributed in the Gulf of Bothnia (GB, triangle), Baltic Proper (BP, circles) and the Skagerrak-Kattegat (SK, squares). Colour of the points is scaled by station-level mean salinity. Inset shows the distribution of all monthly salinity measurements (0–10 m depth) from stations in the three regions. The map was created using R^22^.

To answer these questions, we used two decades of light-microscope based monitoring of nano- and microphytoplankton species (> 2 µm), cell abundance, and biomass, across a broad salinity gradient^22–35^ spanning three sea basins along the Swedish coastline: the low salinity Gulf of Bothnia, the intermediate salinity Baltic Proper, and the high salinity Skagerrak-Kattegat (Fig. 1).

## Results

*Question 1:* Does phytoplankton community structure vary spatially along a broad salinity gradient?

Overall, we analysed 3708 monthly nano- and microphytoplankton community samples from 19 sampling stations over a 19-year timeframe (1999–2017). Across all these monthly samples from the sampling stations, we identified 654 phytoplankton species above 2 µm (see methods for description of species). We used five metrics to describe community structure and answer Question 1 (see Table 1 for full description). Briefly, γ (gamma) biodiversity was measured as species richness and effective number of species (ENS) and refers to the total phytoplankton diversity for each station across all monthly samples. The mean α (alpha) biodiversity, also measured as species richness and ENS, refers to the mean diversity per month for each station. Similarly, mean cell abundance refers to mean cell abundance per month for each station.

**Table 1:** Biodiversity metrics used to characterise phytoplankton community structure and its explanation. We estimated two aspects of biodiversity: species richness and effective number of species (ENS).

Scale	Community structure metric	Definition
**Question 1**		
Station	γ species richness	Total number of species in all monthly samples at a given station rarefied by the number of monthly samples
	γ ENS	Total ENS of all monthly samples at a given station rarefied by the number of monthly samples
	Mean cell abundance	Mean total cell abundance per station across all monthly samples
	Mean α species richness	Mean number of species per station rarefied by number of individuals across all monthly samples
	Mean α ENS	Mean ENS per station rarefied by number of individuals across all monthly samples
**Question 2**		
Monthly sample	Cell abundance	Total cell number in a monthly sample from a given station
	α species richness	Number of species in a monthly sample from a given station rarefied by the number of individuals in the sample
	α ENS	ENS of a monthly sample from a given station rarefied by the number of individuals in the sample

**Figure 2:**
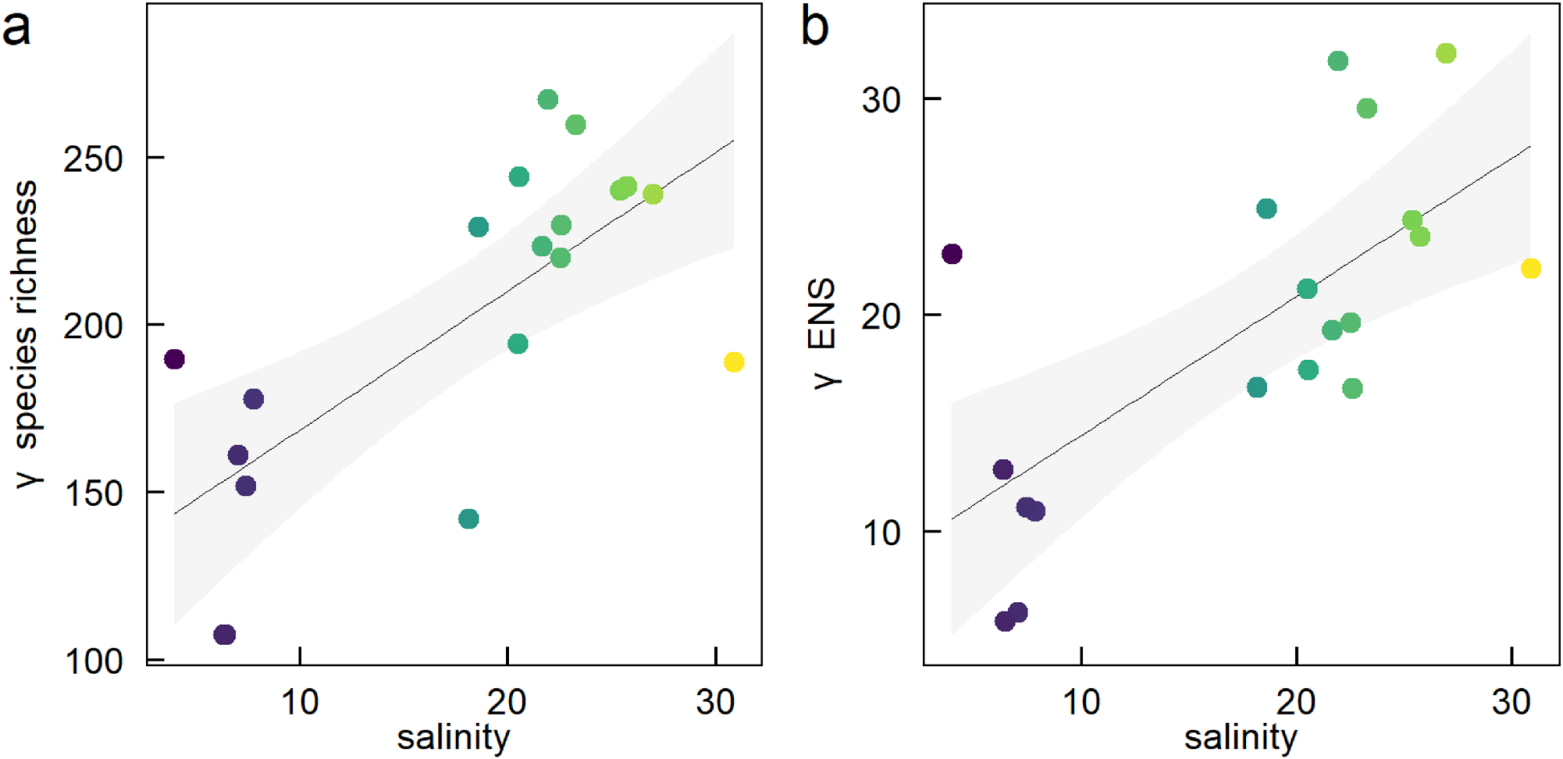
Relationship between mean station-level salinity and (**a**) γ species richness (rarefied at the monthly sample level), and (**b**) γ ENS (rarefied at the monthly sample level). Regression lines are presented with 95% confidence bands. Different colours are different stations with different mean station-level salinities (colours are the same as in Fig. 1).

At the station-scale, phytoplankton community structure varied considerably along the salinity gradient. Both γ species richness and γ ENS increased significantly with salinity (LM: Est.[CI_95%_] = 4.2 [2.1 to 6.2], r^2^ = 0.51 and Est.[CI_95%_] = 0.63 [0.30 to 0.98], r^2^ = 0.48 respectively; Fig. 2). A possible exception is the single station from the Gulf of Bothnia which had higher γ species richness and γ ENS than the relatively higher salinity Baltic Sea stations (Fig. 2). However, without additional data from Gulf of Bothnia, the evidence is limited.

Different phytoplankton groups were differentially represented across sampling stations along the salinity gradient. This variation is due predominately to differential representation of cyanobacteria (mainly filamentous) and diatoms which account for, on average, over 60% of total carbon biomass in monthly samples from all stations (mean ± SD: 0.64 ± 0.11). Cyanobacteria carbon biomass in monthly samples decreased with salinity to almost zero in the high salinity stations (Fig. S1, LM: Est.[CI_95%_] = − 0.058 [− 0.075 to − 0.041], r^2^ = 0.77). In contrast, diatom carbon biomass increased by approximately an order of magnitude for every 16.5 units of salinity (Fig. S1, LM: Est.[CI_95%_] = 0.061 [0.038 to 0.084], r^2^ = 0.66). Dinoflagellates and other autotrophs accounted for 36% of total carbon biomass in monthly samples from all stations (mean ± SD: 0.36 ± 0.11). Dinoflagellate carbon biomass in monthly samples increased with salinity whilst other autotrophs decreased (Fig. S1).

**Figure 3:**
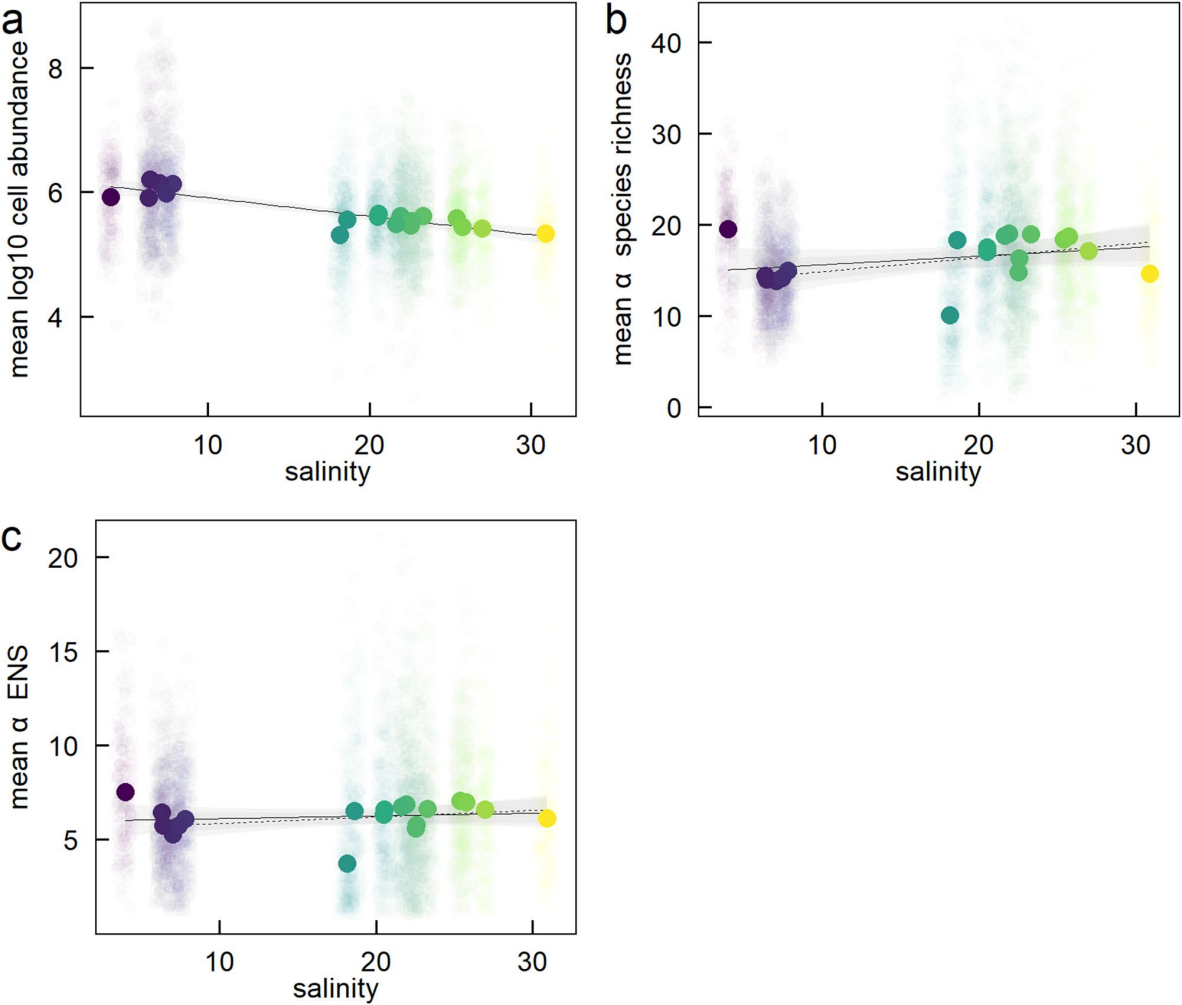
Relationship between mean salinity and (**a**) mean log_10_ cell abundance, (**b**) mean α species richness (rarefied at the individual level) and (**c**) mean α effective number of species (ENS, rarefied at the individual level) at the station-level. Circles are monthly measurements of log_10_ cell abundance, α species richness and α ENS for each station. Filled circles are the mean of all monthly measurements at each station (see Table 1). Regression lines are through the means and are presented with 95% confidence bands. Solid lines include all stations and dashed lines exclude the station from the Gulf of Bothnia (lowest salinity point). Different colours are different stations with different mean station-level salinities (colours are the same as in Fig. 1).

Mean log_10_ cell abundance (log_10_) decreased with mean station-level salinity (LM: Est.[CI_95%_] = − 0.030 [− 0.038 to − 0.022], r^2^ = 0.79) but there was considerable variation around the mean (Fig. 3a). However, the relationship between mean α species richness and mean α ENS with mean-station-level salinity was more complicated. There was a general trend of increasing mean α species richness with salinity (Fig. 3b). However, this relationship was strongly influenced by the lowest salinity station in the Gulf of Bothnia which also had high mean α species richness (Fig. 3b, see green filled circle). Without this station, mean α species richness increased significantly with mean station-level salinity (Fig. 3b, dashed line, LM: Est.[CI_95%_] = 0.16 [0.013 to 0.30], r^2^ = 0.25). Adding second-order terms to the model did not improve model fit (ΔAIC: 1.9; F_17,16_ = 0.0018, P = 0.97). In contrast, there was no relationship between mean α ENS and mean station-level salinity with (LM: Est.[CI_95%_] = 0.014 [− 0.036 to 0.065], r^2^ = 0.02) or without (LM: Est.[CI_95%_] = 0.035 [− 0.015 to 0.085], r^2^ = 0.12) the Gulf of Bothnia station (Fig. 3c, solid and dashed line respectively).

### Question 2: How does phytoplankton community structure vary seasonally, does the seasonal variation change along the salinity gradient, and which environmental variables explain this variation?

**Figure 4:**
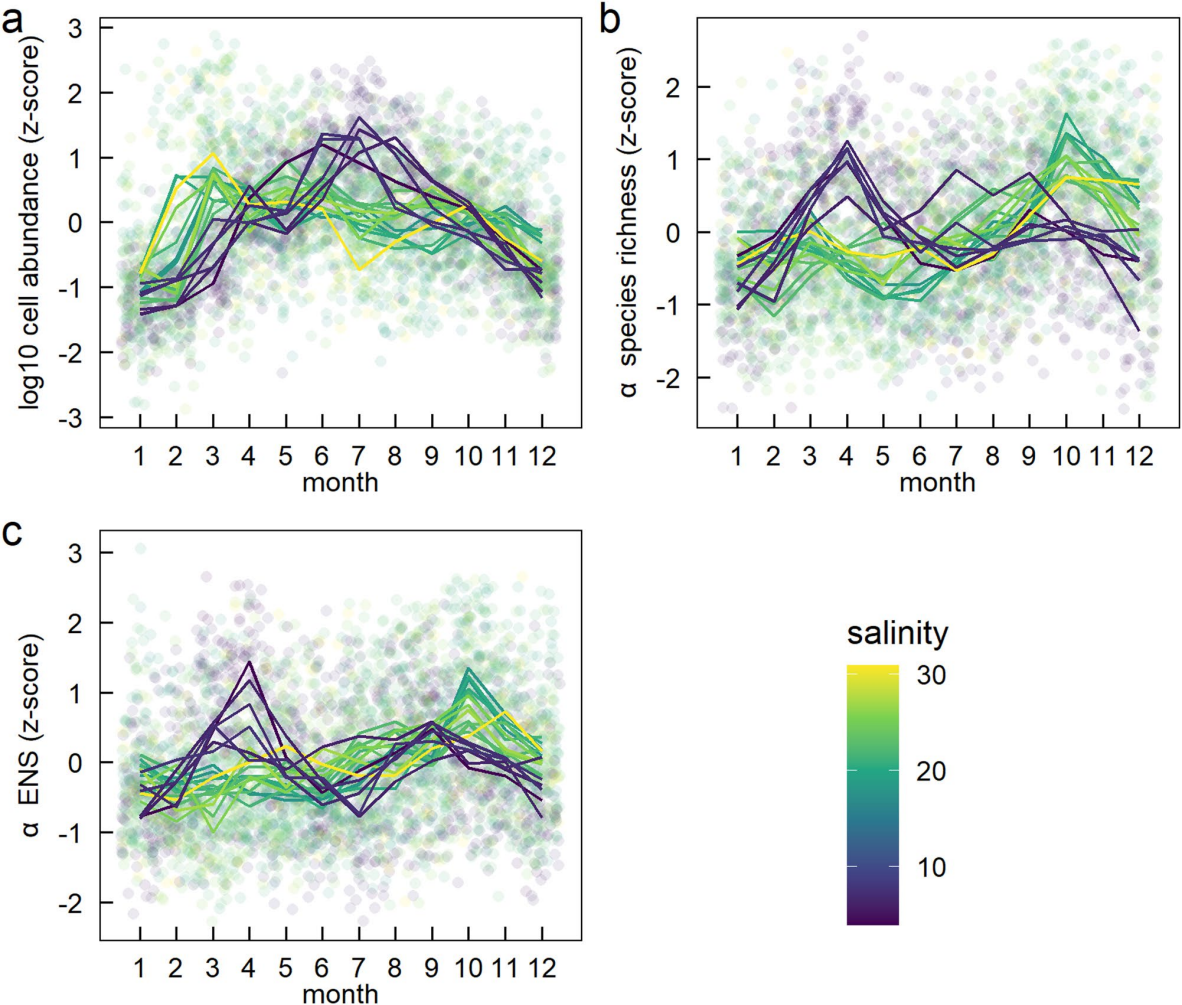
Seasonal variation in (**a**) log10 cell abundance (**b**) α species richness (rarefied at the individual level) and (**c**) α effective number of species (ENS) rarefied at the individual level) by calendar month between years. Faded circles are z-scores relative to the within-year mean and standard deviation for each station. Lines join the mean z-score for each station for each calendar month. Different colours are different stations with different mean station-level salinities.

To answer question 2, we used individual monthly measurements of α biodiversity (species richness and ENS) and cell abundance (see Table 1 for full description). Despite similar seasonal variation in dissolved inorganic nitrogen (DIN), dissolved inorganic phosphorus (DIP) and sea surface temperature (SST) at the stations, seasonal variation in phytoplankton community structure differed across the salinity gradient. We visualised seasonal variation in community structure using deviation from the within-year mean for each month based on z-scores. At the high salinity stations, log_10_ cell abundance was highest in early spring (Fig. 4a). However, in the less saline stations from the Gulf of Bothnia and Baltic Sea, log_10_ cell abundance was highest in spring and summer (Fig. 4a). Seasonal variation in α species richness and α ENS showed an opposite pattern to cell abundance (Fig. 4b,c). In stations from the high salinity Skagerrak-Kattegat, α species richness peaked during autumn and early winter whilst α species richness in the Gulf of Bothnia and Baltic Proper was highest during spring.

These patterns were confirmed by explicitly examining how the month in which log10 cell abundance, α species richness and α ENS peaked changed along the salinity gradient. The mean annual peak month in log10 cell abundance was earlier by one and a half months for every 10-unit increase of salinity (Fig. S2, LM: Est.[CI_95%_] = − 0.15 [− 0.22 to − 0.07], r^2^ = 0.51). In contrast, the mean annual peak month of α species richness increased with salinity (Fig. S2, LM: Est.[CI_95%_] = 0.29 [0.20 to 0.37], r^2^ = 0.74) and similarly for α ENS (Fig. S2 LM: Est.[CI_95%_] = 0.24 [0.16 to 0.31], r^2^ = 0.71).

In addition to differences in the timing of the peaks, the magnitude of within-year variation, measured as the range among months within a year also varied with salinity. Specifically, the within-year range in log10 cell abundance decreased with salinity (Fig. S3, LM: Est.[CI_95%_] = − 0.02 [− 0.033 to − 0.0003], r^2^ = 0.21). The opposite pattern was found for α species richness and α ENS: the range among months increased with salinity (Fig. S1 LM: Est.[CI_95%_] = 00.38 [0.26 to 0.50], r^2^ = 0.71 and Fig. S3, LM: Est.[CI_95%_] = 0.13 [0.058 to 0.20], r^2^ = 0.47 respectively). Thus, lower cell abundance variation corresponds to higher α species richness and α ENS within a year.

**Figure 5:**
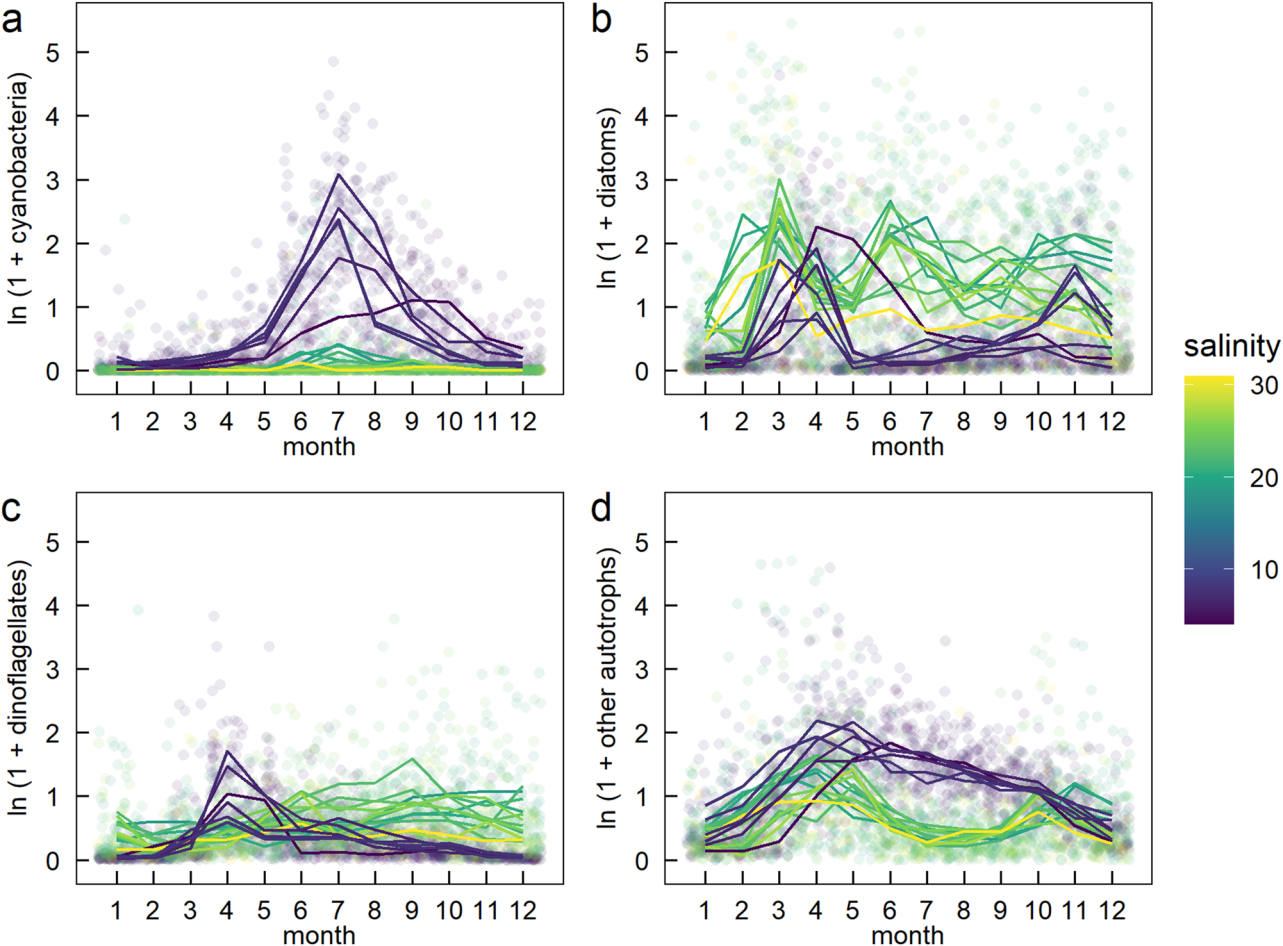
Seasonal variation in ln (1 + carbon biomass), (µ g L^−1^) of (**a**) cyanobacteria, (**b**) diatoms, (**c**) dinoflagellates and (**d**) other autotrophs by calendar month. Faded circles are monthly values for each sampling station across years. Lines join the mean ln(1 + carbon biomass) for each station for each calendar month. Different colours are different stations with different mean station-level salinities.

Seasonal variation in the identity of the dominant phytoplankton group varied considerably across the salinity gradient. At low salinity stations in the Gulf of Bothnia and Baltic Sea, there is a strong cyanobacteria peak in late summer and autumn and a corresponding diatom dip (compare Fig. 5a,b). In contrast, the proportion of cyanobacteria biomass in higher salinity stations remains low throughout the year (Fig. 5a). The higher salinity stations are dominated by diatoms all year round and seasonal variation is less pronounced than for cyanobacteria (Fig. 5a,b).

Seasonal variation in DIN, DIP, and SST was strong at all stations across the salinity gradient and showed similar seasonal cycles. SST peaked predictably during summer (Fig. S4). DIN followed the opposite trend, peaking in the autumn and winter in all the regions and DIP followed a similar seasonal trend to DIN (Fig. S4). Seasonal variation in silicate was less pronounced than the other variables (except for in the Gulf of Bothnia) but, like DIN and DIP, also peaked in autumn–winter (Fig. S4).

We then examined which environmental variables (SST, ln DIN, ln DIP, $\sqrt[2]{\text{silicate}}$ and mean station-level salinity) best explained seasonal variation in log10 cell abundance, $\sqrt[2]{{\alpha {\text{ species richness}}}}$ and $\sqrt[2]{{\alpha {\text{ ENS}}}}$. Among these variables, ln DIN and SST and ln DIN and ln DIP were highly correlated (Pearson’s r [CI_95%_]: − 0.60 [− 0.62 to − 0.58] and 0.63 [0.61 to 0.65] respectively). Given this, we focused on ln DIN, $\sqrt[{2}]{{{\text{silicate}}}}$ and mean station-level salinity as predictor variables.

Seasonal variation in log10 cell abundance was best explained by a model with ln DIN, $\sqrt[{2}]{{{\text{silicate}}}}$, mean station-level salinity and the interaction between ln DIN and mean station-level salinity (LMM: AIC weight > 0.99, Table 2). This model explained almost 40% of the variation (conditional r^2^ = 0.37), most of which was due to the fixed effects (marginal r^2^ = 0.33). This model showed that log10 cell abundance was negatively related to ln DIN (LMM: Est.[CI_95%_]: − 0.38 [− 0.41 to − 0.34]) but only at stations with low mean salinity as evidenced by the positive ln DIN and mean station-level salinity interaction (LMM: Est.[CI_95%_]: 0.014 [0.012 to 0.016]). At stations with high salinity like those in the Skagerrak-Kattegat, log10 cell abundance was not related to ln DIN. In addition, log10 cell abundance was negatively related to both $\sqrt[{2}]{{{\text{silicate}}}}$ and mean station-level salinity (Table 3).

**Table 2:** Best models (lowest AIC) explaining seasonal variation in three response variables: log10 cell abundance, $\sqrt[2]{{\alpha {\text{ species richness}}}}$ and $\sqrt[2]{{\alpha {\text{ ENS}}}}$. Best models were determined by AIC (ΔAIC > 2). For each response variable, all possible combinations of ln DIN, $\sqrt[2]{{{\text{silicate}}}}$, and the interaction between ln DIN and mean station-level salinity were used. Mean station-level salinity was included in all models. This resulted in six models in total. When no clear model emerged, all equivalent models are reported.

Model	Marginal r^2^	Conditional r^2^	AIC	AIC weight
**log10 cell abundance**				
(int.) + ln DIN + salinity + $\sqrt[2]{{{\text{silicate}}}}$ + ln DIN:salinity	0.33	0.37	6131	> 0.99
$$\sqrt[{\mathbf{2}}]{{{\mathbf{\alpha }}\,{\mathbf{species }}\,{\mathbf{richness }}}}$$				
(int.) + ln DIN + salinity + $\sqrt[2]{{{\text{silicate}}}}$	0.022	0.18	7523	0.38
(int.) + ln DIN + salinity	0.021	0.18	7524	0.30
(int.) + ln DIN + salinity + ln DIN:salinity	0.021	0.18	7525	0.16
(int.) + ln DIN + salinity + $\sqrt[2]{{{\text{silicate}}}}$ + ln DIN:salinity	0.022	0.18	7525	0.15
$$\sqrt[{\mathbf{2}}]{{{{\varvec{\upalpha}}}\,{\mathbf{ ENS }}}}$$				
(int.) + ln DIN + salinity + $\sqrt[2]{{{\text{silicate}}}}$ + ln DIN:salinity	0.013	0.10	6286	0.54
(int.) + ln DIN + salinity + ln DIN:salinity	0.012	0.093	6286	0.44

**Table 3:** Estimated model coefficients for the best model explaining seasonal variation in log10 cell abundance (see Table 2).

Response	Parameter	Estimate	Lower CI_95%_	Upper CI_95%_
log10 cell abundance	(intercept)	6.6	6.4	6.9
	ln DIN	− 0.37	− 0.41	− 0.34
	salinity	− 0.034	− 0.047	− 0.027
	$\sqrt[{2}]{{{\text{silicate}}}}$	− 0.13	− 0.16	− 0.098
	ln DIN:salinity	0.014	0.012	0.016

Unlike log10 cell abundance, seasonal variation in $\sqrt[2]{{\alpha {\text{ species richness}}}}$ and $\sqrt[2]{{\alpha {\text{ ENS}}}}$ was poorly explained by ln DIN, $\sqrt[{2}]{{{\text{silicate}}}}$ and mean station-level salinity. For $\sqrt[2]{{\alpha {\text{ species richness}}}}$, no clear best model emerged, and, for all models, the fixed effects explained less than 2.5% of the variation (Table 2). Similarly, for $\sqrt[2]{{\alpha {\text{ ENS}}}}$, no best model emerged and, in all models tested, the fixed effects explained less than 1.5% of the variation. Using 1-month time-lagged ln DIN and $\sqrt[{2}]{{{\text{silicate}}}}$ slightly improved model fits for $\sqrt[2]{{\alpha {\text{ species richness}}}}$ and $\sqrt[2]{{\alpha {\text{ ENS}}}}$ (see supplementary material for more details). However, these models still explained less than 6% of the variation and, therefore, did not alter our conclusions (Table S1).

## Discussion

### Question 1: Does phytoplankton community structure vary spatially along a broad salinity gradient?

We found considerable spatial variation in broad-scale phytoplankton species richness across the salinity gradient. Specifically, both γ (gamma) species richness and γ effective number of species (ENS) were almost twice as high in the highest compared to the lower salinity stations. This may be due the greater impact of currents and stratification in the Skagerrak-Kattegat compared to the Baltic Sea^23^ which may enhance species richness of plankton due to, for example, advection from the North Sea. Including water transport in ecological models was recently demonstrated important in order to attain a high diversity of phytoplankton^24^. Mean α (alpha) species richness and α ENS, which represent the diversity of an interacting ecological assemblage, also increased with salinity but the increases were more modest. Moreover, this increase in species diversity occurred despite a decrease in mean cell abundance with salinity. Taken together this suggests a potential ecological limit to species diversity for interacting phytoplankton assemblages at a given point in time despite considerable differences in γ diversity in space.

Despite the general increase in species diversity with salinity, the station with the lowest salinity (our only station from the Gulf of Bothnia, see Fig. 1) also had high γ and α species richness and ENS relative to the intermediate salinity stations. This is partially in line with the hypothesis that species diversity of aquatic groups should peak at high and low salinities on a gradient from seawater to freshwater^16^. Indeed, Olli et al.^18^ examined phytoplankton communities in the brackish Baltic Sea and Chesapeake Bay and their results support this hypothesis at local and regional scales. However, as we only had data from a single station from the low salinity Gulf of Bothnia, our results are not conclusive.

Despite a decrease in mean cell abundance with salinity (Fig. 3), mean carbon biomass increased with salinity (Fig. S1). This is likely explained by differences in the cell size of dominating phytoplankton groups. The overall highest carbon biomass is by diatoms, and they dominate in the high salinity stations. In contrast, the low salinity stations are dominated by cyanobacteria and other autotrophs, e.g., flagellates, which generally are smaller in their cell size as compared to diatoms.

### Question 2: How does phytoplankton community structure vary seasonally, does seasonal variation change along the salinity gradient, and which environmental variables explain this variation?

Seasonal variation in cell abundance varied considerably along the salinity gradient. With higher salinity, cell abundance peaked earlier in the year. At stations with higher salinity (Skagerrak-Kattegat region) cell abundance peaked during spring. The higher salinity stations were dominated by diatoms, which reached the highest cell abundance and carbon biomass in spring when nutrients are available after winter. In contrast, at stations with lower salinity (Baltic Proper and Gulf of Bothnia regions), cell abundance peaked in summer. This was likely due to the high abundance of filamentous cyanobacteria at lower salinity stations, forming dense blooms when temperatures are stimulating their growth^25^. Moreover, filamentous cyanobacteria can bypass nitrogen limitation by fixing atmospheric nitrogen dissolved in the water which gives them an advantage over other phytoplankton groups in low DIN:DIP ratios^9,26^. However, cyanobacteria are strongly limited by salinity and are therefore mostly absent in the high salinity stations. In contrast the within-year variation in cell abundance was higher in the low salinity stations in comparison with the high salinity stations. This was likely due to the summer blooms of filamentous cyanobacteria and low winter cell abundances in the lower salinity stations.

Seasonal variation in α species richness and α ENS showed similar patterns but did not match with seasonal variation in cell abundance. Both α species richness and ENS peaked during the fall in high salinity stations and were thus uncoupled from the spring cell abundance peak. This is in line with Olofsson et al.^27^ who reported a highly diverse phytoplankton community during August and September in the high salinity Skagerrak-Kattegat which was dominated by a combination of diatoms and dinoflagellates. These late summer to early fall communities are formed during low nutrient availability, but relatively high temperatures and light availability, which supposedly stimulates high species diversity. In contrast, α species richness and ENS peaked in spring at lower salinity stations from the Baltic Proper and Gulf of Bothnia and were uncoupled from the summer peak in cell abundance. This is likely due to the dense blooms of filamentous cyanobacteria during summer in the low salinity stations which results in high cell abundance but low diversity. Therefore, α species richness instead peaked in the low salinity stations during spring when the community consisted of a mixture of phytoplankton groups (Figs. 5 and S2). Spring blooms occurred later in the Baltic Sea as compared to the high salinity stations, something that may also affect the composition and diversity of the phytoplankton communities. In addition, we observed a unimodal relationship between cell abundance and diversity (Fig. S5), as has previously been observed for primary productivity and diversity^28–30^. This also relates to high summer cell abundance in the low salinity stations and early spring blooms dominated by fast-growing diatoms in the high salinity stations, resulting in less diverse communities.

Seasonal variation in species diversity was poorly explained by environmental variation. This may be somewhat surprising given that phytoplankton diversity varies spatially with temperature over large scales^31^. Several factors may explain our result. First, even though we used monthly samples, this is still a low temporal resolution for phytoplankton communities which can turnover rapidly^32^. Second, the environmental measurements were taken at the same time as the community sample. Even if phytoplankton communities respond rapidly to environmental changes, it is possible that diversity would be related to the environmental condition at a previous time point. However, including time-lagged variables in our analyses did not result in a higher amount of explained variation (Table S1). Third, in all regions there was considerable variation in the dominant phytoplankton group throughout the season and different groups of phytoplankton species may respond differently to environmental variation^33^. In addition, the lack of explanation for species variation by environmental parameters can also be due to interactions with surrounding organisms. We did not consider predation by micro- (e.g. ciliates and dinoflagellates) and meso-zooplankton (mainly copepods, cladocerans, appendicularians). Predation by these groups is known to have strong impacts on both the abundance and composition of the phytoplankton community^34,35^. In addition, heterotrophic bacteria are known to live in close relationships with phytoplankton communities^36–38^. These heterotrophic bacteria also show strong seasonality in both abundance and productivity in the Baltic Sea^39^ and thus could also explain the temporal variation in species richness. How interactions among phytoplankton and other key planktonic groups affect phytoplankton diversity warrants further examination.

### Caveats

Since our estimates of phytoplankton community diversity focused on nano- and microphytoplankton > 2 μm, the smallest phototrophic picoplankton were not included in our dataset. However, since phototrophic picoplankton have limited niche overlap with larger phytoplankton due to variation in nutrient uptake, light absorption, and growth rates^40^, it is likely that the drivers of phototrophic picoplankton differs to those of larger phytoplankton. Specifically, due to their large surface area to volume ratio, picoplankton has an advantage over larger phytoplankton under nutrient-limited conditions and high grazing pressure. Therefore, picoplankton rarely dominate the phytoplankton communities. Specifically, picoplankton comprised, on average, 30% of the chlorophyll biomass across 29 stations in the Baltic Sea and the North Sea^41^ and between 0 and 52% in the Kiel Bight, with the highest proportions observed during summer^42^. Recent studies have shown that species diversity estimates based on metabarcoding (also including picoplankton) are higher than estimates based on light microscopy but that direct conversions to species can be challenging^43,44^. Therefore, a combination of microscopy and genetic methods likely will more fully capture total phytoplankton community structure.

### Conclusions

Understanding the dynamics of phytoplankton abundance and biodiversity in coastal ecosystems is of high interest due to their central role in biogeochemical cycles. Our study provides insights into spatial and temporal variation in phytoplankton community structure and abundance along a broad salinity gradient. By simultaneously examining grazing, nutrients, and water transport, a recent ecosystem model demonstrated that phytoplankton diversity is sensitive to all these factors^24^. Here, we combined multiple environmental factors such as nutrients, temperature, and salinity, but could not identify the drivers of the seasonal variation in phytoplankton diversity. Including biotic factors may thus be key to understand spatial and temporal variation in phytoplankton diversity and community structure. Only by expanding our knowledge base will we be able to predict the consequences of global change for biodiversity and ecosystem functioning.

## Materials and methods

### Monitoring data and study area

The Swedish National Marine Monitoring Program includes monthly tube sampling of nano- and microphytoplankton abundance (> 2 µm) and water collection for chemical and physical parameters. For the Skagerrak-Kattegat, data from regional monitoring using identical methods were included. This database includes phytoplankton cell abundances (cells L^-1^) and carbon biomass (μg L^−1^) based on cell abundance^45^ and cell sizes^46^, calculated based on Menden-Deuer and Lessard^47^. The nanoplankton (2–20 µm) were, in general, identified to species or genus level for diatoms, dinoflagellates, and filamentous cyanobacteria, while e.g., haptophytes and cryptophytes were rather identified to genus or order level. The microplankton (20–200 µm) were well identified to species or genus level. For organisms identified to the same genus level they are pooled together in the analyses, e.g., to *Chaetoceros* spp. rather than *Chaetoceros* sp.1 and sp. 2. While taxa were not always identified to the species level, we refer to taxon richness and composition as species richness and composition throughout the article. We considered this reasonable because our metrics of community structure calculated at genus or species levels correlate strongly (Pearson’s r > 0.90, Fig. S6 and Table S2). The smallest phytoplankton, the phototrophic picoplankton (< 2 µm) are not included due to restrictions of the method. The standardised species list with cell volumes of the HELCOM Phytoplankton Expert Group was used (available at https://ices.dk/marine-data/Documents/ENV/PEG_BVOL.zip). These data are hosted by the Swedish National Oceanographic Data Centre at the Swedish Hydrological and Meteorological Institute and are available via open access at https://sharkweb.smhi.se.

For this study, we compiled time-series of monthly monitoring data of phytoplankton cell abundances (cells L^-1^) and physiochemical water properties (surface salinity (0–10 m), sea surface temperature (°C, 0–10 m, SST), dissolved inorganic phosphate (DIP), dissolved inorganic nitrogen (nitrate, nitrite, and ammonium, DIN), and silicate) from 19 sampling stations that span a wide salinity gradient from three well-known regions on the Swedish coastline: Gulf of Bothnia (n = 1), Baltic Proper (n = 5) and the Skagerrak-Kattegat (n = 13), (Fig. 1, see Table S3 for GPS coordinates). Inorganic nutrients sampled by the monitoring programme were measured according to HELCOM^48^. These time-series were collected between 1999 and 2017. While most samples were monthly, there were several cases where sampling at a given station occurred more than once in a given month. In these cases, we took the mean abundance of each species sampled in that month and the mean of the physio-chemical variables. In addition, the time-series of monthly monitoring data have varying levels of completeness. Given that we were predominately interested in seasonal trends in phytoplankton community structure, we required data from sites that had adequate numbers of monthly samples in a year. Thus, these 19 stations were selected because they had more 10 years of data where eight or more months were sampled (see Table S4).

In addition to the phytoplankton cell abundance and physiochemical water property data, we also used carbon biomass (μg L^−1^) data. The carbon biomass of phytoplankton was aggregated into four groups (cyanobacteria, diatoms, dinoflagellates, and flagellates and other autotrophic organisms > 2 µm, using Plankton Toolbox (version 1.3.1)^49^. These data were less complete than the phytoplankton cell abundance and physiochemical data. One of the 19 stations had very little carbon biomass data and therefore was excluded (see Table S4 for an overview of different data subsets). Nonetheless, these data serve as a complement to understanding patterns in the phytoplankton cell abundance and diversity.

## Data analyses and statistics

### Question 1: Does phytoplankton community structure vary spatially along a broad salinity gradient?

To address this question, we first examined how phytoplankton community structure varied along the natural salinity gradient at the station-scale (all monthly samples at sampling station). At the station-scale we calculated the number of phytoplankton species observed across all monthly samples: the temporal γ (gamma) species richness, and the temporal γ (gamma) effective number species (ENS), (γ species richness and γ ENS hereafter, Table 1). The ENS weights species by their relative abundance and is preferred to classical Shannon entropy because it obeys the doubling principle. Because of this abundance weighting, it can be interpreted as the total number of common species^50^. Both were rarefied at the monthly sample-scale down to the station with the fewest monthly samples (n = 133). This was done by randomly drawing 500 replicate samples with replacement of 133 monthly samples and calculating the mean species richness and ENS of the 500 replicates. We then used simple linear regression to examine the relationships between mean salinity at each station (mean across all monthly samples) and (i) γ species richness and (ii) γ ENS.

We calculated three aspects of local community structure: mean cell abundance (cells L^−1^), mean α (alpha) species richness and mean α (alpha) ENS. In contrast to γ species richness and γ ENS, these metrics reflect the average community structure in any given month and thus are akin to the mean condition in an interacting phytoplankton assemblage. To reduce bias due to differences in overall cell abundance between samples, we rarefied α species richness and α ENS to the equivalent of the monthly sample with the lowest total cell abundance (iNEXT function, *iNEXT* package) ^50,51^. As previously, we used simple linear regression to examine the relationship between mean salinity at each station and the mean (i) log10-transformed cell abundance, (ii) mean α species richness and (iii) mean α ENS. For α species richness and α ENS, the single station from the Gulf of Bothnia poorly fit the linear relationship. Thus, we tested whether adding second-order polynomial terms improved model fit using AIC (Δ > 2) and F-tests. In addition, we fit the simple linear regression models with and without the outlier and compared the results.

We then used the available carbon biomass data (see Table S4) to examine the proportional representation of four major phytoplankton groups: diatoms, cyanobacteria, dinoflagellates and other autotrophs. To do this, we calculated the proportion of biomass from each phytoplankton group in each monthly sample and used these values to calculate an overall mean for each station, for each phytoplankton group. The mean proportion of each phytoplankton group was then regressed against mean salinity at each station using simple linear regression. For all simple linear regression models, we checked assumptions of normality and equal variance using graphical analyses of the residuals.

### Question 2: How does phytoplankton community structure vary seasonally, does the seasonal variation change along the salinity gradient, and which environmental variables explain this variation?

First, to understand seasonal variation in different environmental variables, we graphically explored seasonal variation in dissolved inorganic nitrogen (DIN), dissolved inorganic phosphorous (DIP), sea surface temperature (SST) and silicate. Second, we examined seasonal patterns of phytoplankton community structure among stations along the salinity gradient. For this, we used four complementary approaches. First, for log10-cell abundance, α species richness and α ENS (see Table 1 for definitions), we calculated z-scores based on the within-year mean and standard deviation for each station to assess how different months deviated from the within-year expectation. These z-scores were examined graphically. Second, we quantitatively estimated the month in which log10-cell abundance, α species richness and α ENS peaked. To do this, for each year at each station, we recorded the month with the highest value for each of these three community structure metrics. Then, at each station, we used a circular mean (mean.circular function, *CircStats* package) to calculate the mean month where peaks occurred for each community structure metric (0 represents January and 11 represents December). Third, we calculated the magnitude of within-year variation in log10-cell abundance, α species richness and α ENS using the within-year range (within-year max − within-year min). We regressed the circular mean peak month and within-year range of log10-cell abundance, α species richness and α ENS against mean salinity at each station. The assumptions of normality and equal variance for these simple linear regression models were also checked using graphical analyses of the residuals. Finally, to understand seasonal variation in the abundance of different phytoplankton groups, we graphically explored seasonal variation in ln-transformed carbon biomass of diatoms, cyanobacteria, dinoflagellates and other autotrophs.

Finally, we examined how the five available environmental variables: dissolved inorganic nitrogen (DIN), dissolved inorganic phosphorus (DIP), sea surface temperature (SST), silicate and mean station-level salinity were related to log10-cell abundance, α species richness and α ENS using linear mixed models. Both DIN and DIP were ln-transformed and silicate was square-root transformed to improve variable distributions. Among these variables ln DIN and SST and ln DIN and ln DIP were highly correlated (Pearson’s r [CI_95%_]: − 0.60 [− 0.62 to − 0.58] and 0.63 [0.61 to 0.65] respectively) which lead to high variance inflation (variance inflation factors > 10). Given these constraints, we focused on ln DIN, $\sqrt[{2}]{{{\text{silicate}}}}$ and mean station-level salinity as predictor variables. In addition, α species richness and α ENS were square root transformed to stabilise variance.

For log10 cell abundance, α species richness and α ENS, we built a global model that included ln DIN, $\sqrt[{2}]{{{\text{silicate}}}}$, mean station-level and the interaction between mean station-level salinity and ln DIN as predictor variables, with station as a random intercept. We then used AIC to compare all subsets of this global model whilst retaining mean station-level salinity and station as a random intercept. We chose this random effect structure after following Zuur et al.’s^52^ guidelines. First, we fit the global model using generalised least squares and restricted maximum likelihood with only the fixed effects (gls function, *nmle* package). Second, we fit the global model using restricted maximum likelihood with all the fixed effects and the random intercept for station (lmer function, *lme4* package). These models were compared with AIC. For all three community structure variables, the global model with the random intercept for station had a lower AIC (Δ > 2) and thus was used.

We then compared all subsets of the global model using AIC and AIC weights. Models were considered superior if the AIC was two or more units lower. We present only the lowest AIC model(s) in the main text. In addition, we assessed model fit using marginal and conditional *r*^2^ values (*r*^2^_m_ and *r*^2^_c_, rsquared function, *piecewiseSEM* package)^53^ and calculated confidence intervals around fixed effects using Wald approximations. Assumptions of residual normality and homoscedasticity were assessed graphically according to the guidelines of Zuur et al.^52^ and Harrison et al.^54^.

Our phytoplankton community structure and environmental variable measurements are given on a monthly timescale. Therefore, it is possible that there is a temporal mismatch. For example, phytoplankton community structure in one month may reflect the environmental conditions of the previous month. We tested this possibility by repeating our modelling procedure but with ln DIN and $\sqrt[{2}]{{{\text{silicate}}}}$ lagged by 1 month. Therefore, community structure measurements in one month (e.g. February 2011) were related to ln DIN and $\sqrt[{2}]{{{\text{silicate}}}}$ in the previous month (i.e. January 2011). We then compared models using unlagged variables to those with lagged variables. For this analysis, we excluded data points without a value for the environmental variables in the previous months. To maintain comparability, both analyses (unlagged and lagged variables) used this slight subset of the original dataset (see Table S4 for an overview).

All statistical analyses were conducted, and data plotted, in R ver. 4.0.2^22^. Additional packages used were *tidyverse* (data manipulation and visualisation), *iNext* (rarefied species richness, effective number of species, individual-based rarefaction), *lme4* and *nmle* (linear mixed effects models), *piecewiseSEM* (marginal and conditional *r*^2^), *vegan* (rarefaction and diversity indices), and *CircStats* (circular mean).

## Data availability

Data are available from authors upon request.

## Supplementary information

Supplementary Information 1.
