# Supplementary material for: Large seasonal and spatial variation in nano- and microphytoplankton diversity along a Baltic Sea—North Sea salinity gradient
Source: Sci Rep. 2020 Oct 19;10:17666. doi: 10.1038/s41598-020-74428-8 (PMC7572517; doi:10.1038/s41598-020-74428-8)
Supplement: Supplementary file 1 — Supplementary Information 1. [file 41598_2020_74428_MOESM1_ESM.pdf]

Supplementary material - Additional figures, tables, and text

Large seasonal and spatial variation in nano- and microphytoplankton diversity along a Baltic Sea – North Sea salinity gradient

Malin Olofsson[a,+,1,*], James G. Hagan[b,c,1], Bengt Karlson[a], Lars Gamfeldt[b,c,d]

[a]Research and Development, Oceanography, Swedish Meteorological and Hydrological Institute, Sven Källfelts gata 15, SE 426 71, Västra Frölunda, Sweden

[b]Department of Marine Sciences, University of Gothenburg, Gothenburg, Sweden

[c]Gothenburg Global Biodiversity Centre, Box 461, SE-40530, Gothenburg, Sweden

[d]Centre for Sea and Society, Box 260, SE-40530, Gothenburg, Sweden

[+]Currently at: Swedish University of Agricultural Sciences, Department of Aquatic Sciences and Assessment, Uppsala, Sweden

[1]Both authors contributed equally to this work

*Corresponding author: E-mail: malin.olofsson@slu.se. Postal address: Malin Olofsson, Department of Aquatic Sciences and Assessment Box 7050, 750 07 Uppsala, Sweden

Email addresses: Olofsson (malin.olofsson@slu.se), Hagan (james_hagan@outlook.com), Karlson (bengt.karlson@smhi.se), and Gamfeldt (lars.gamfeldt@marie.gu.se).

Results and Discussion

Variation in the biomass of different phytoplankton groups at stations with different mean salinity levels

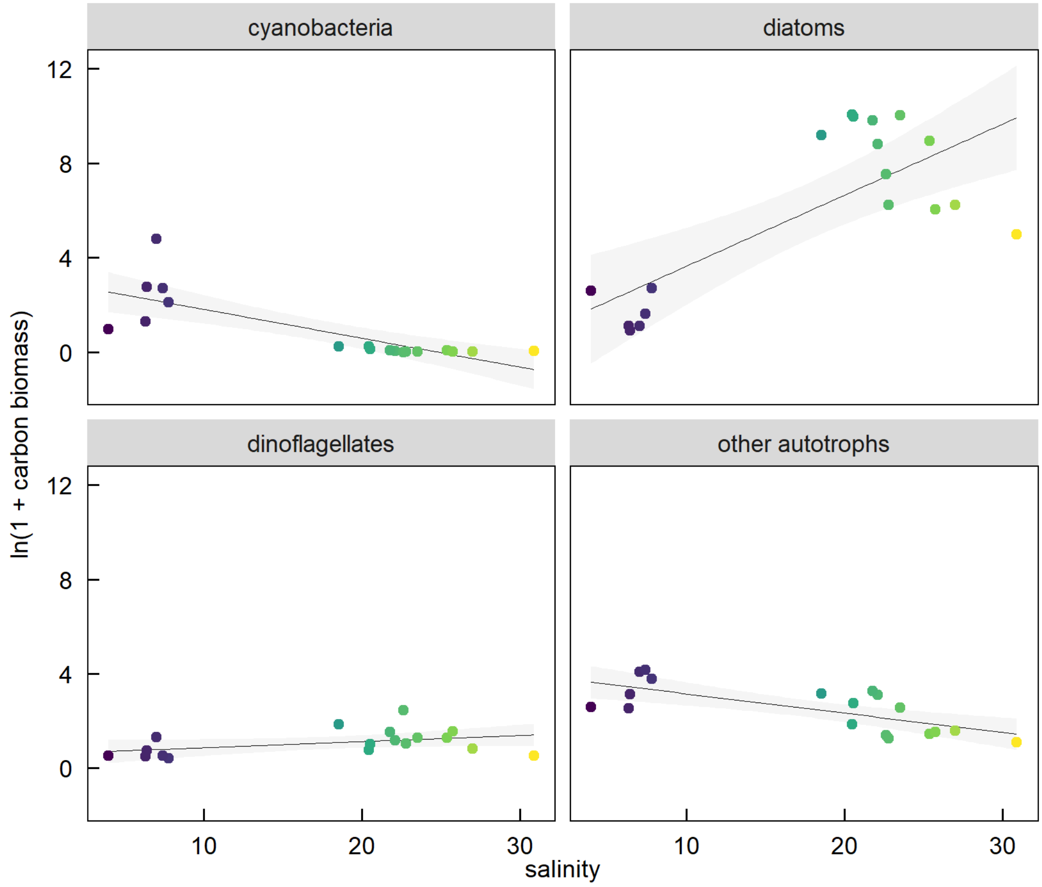

Figure S1. Relationship between mean station-level salinity and mean ln(1 + carbon biomass), (μ g L^{-1}) of cyanobacteria, diatoms, dinoflagellates and other autotrophs of all monthly samples at the different stations (circles). Regression lines are through the station-level means and are presented with 95% confidence bands. Different colours are different stations with different mean station-level salinities (colours are the same as in Fig. 1).

Variation in the annual range in community structure and the timing of peaks among stations across the salinity gradient

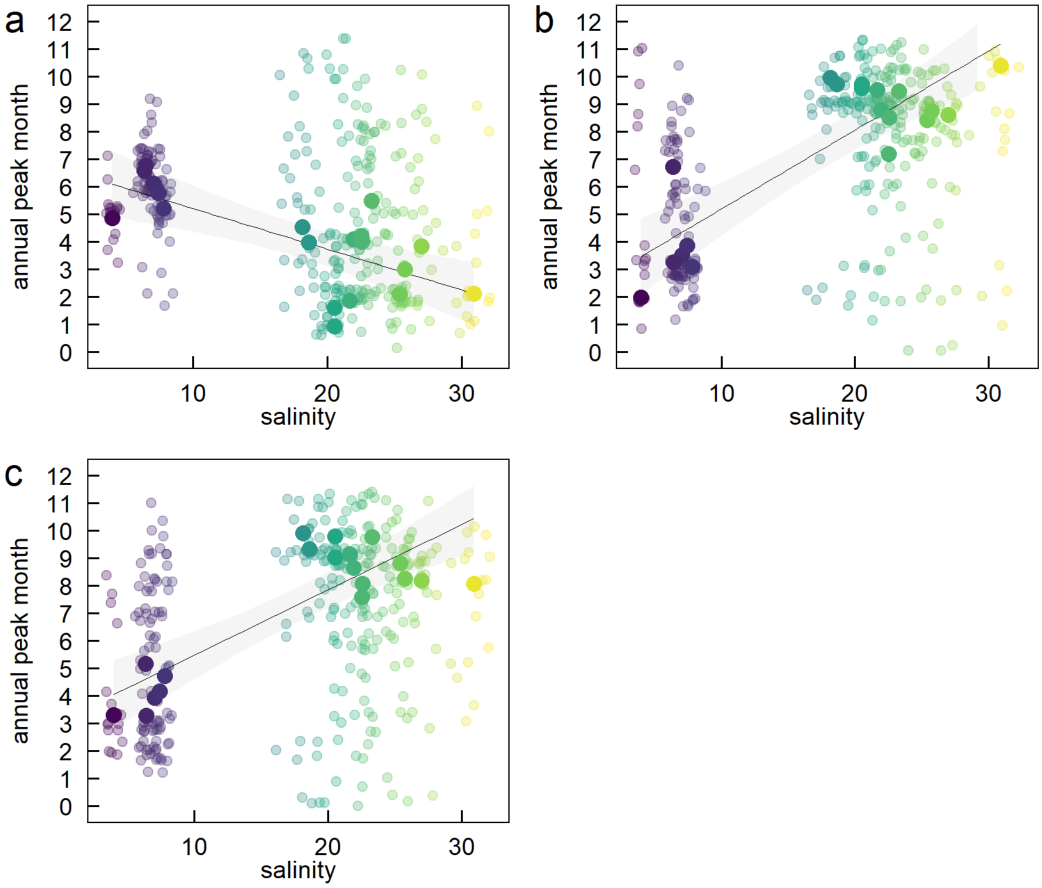

Figure S2. The relationship between mean station-level salinity and mean month of the peak in (a) log10 abundance, (b) α species richness (c) α ENS. Large filled circles are the means of all yearly annual peak month values at each station. Small, faded circles are values for each year. Regression lines are through the means and are presented with 95% confidence bands. Different colours are different regions: Gulf of Bothnia (green), Baltic Proper (purple) and the Skagerrak-Kattegat (yellow). Different colours are different stations with different mean station-level salinities (colours are the same as in Fig. 1).

Variation in the annual range in diversity metrics among stations across the salinity gradient

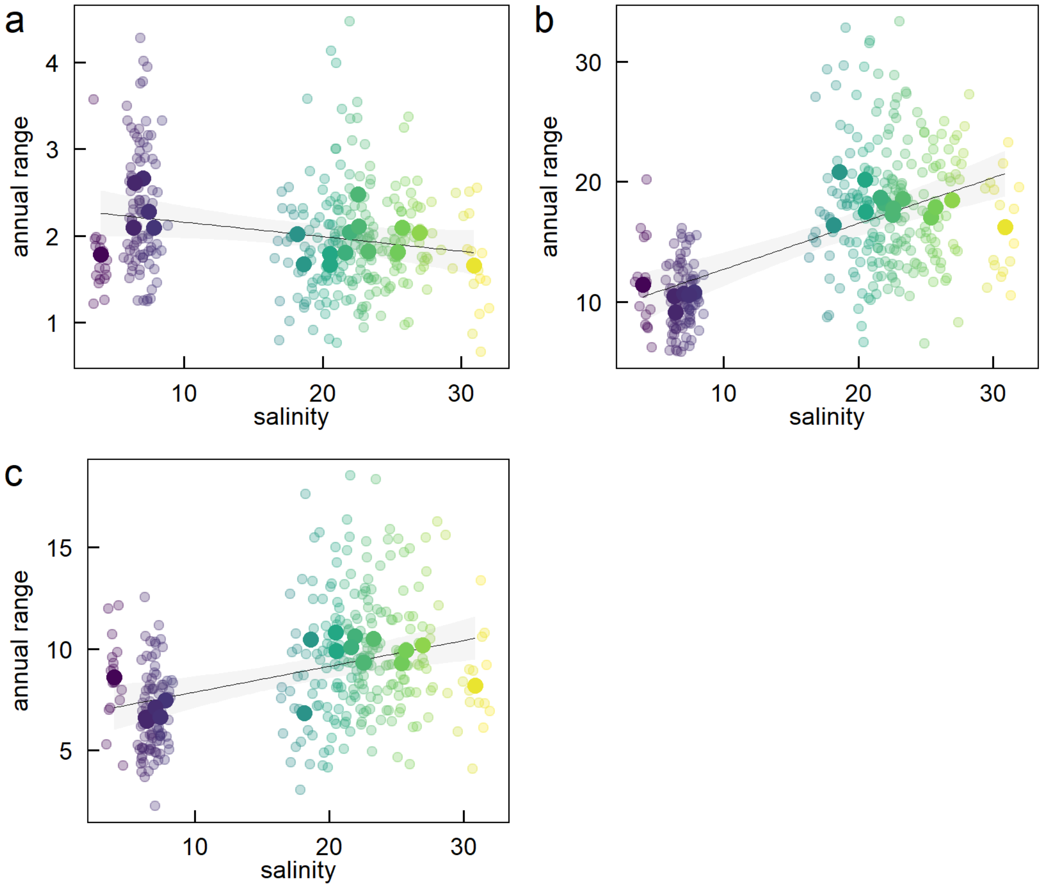

Figure S3. The relationship between mean station-level salinity and the within-year range (maximum-minimum) of (a) log10 abundance, (b) α species richness (c) α ENS. Large filled circles are the means of all yearly ranges at each station. Small, faded circles are values for each year. Regression lines are through the means and are presented with 95% confidence bands. Different colours are different stations with different mean station-level salinities (colours are the same as in Fig. 1).

Seasonal environmental variation across stations

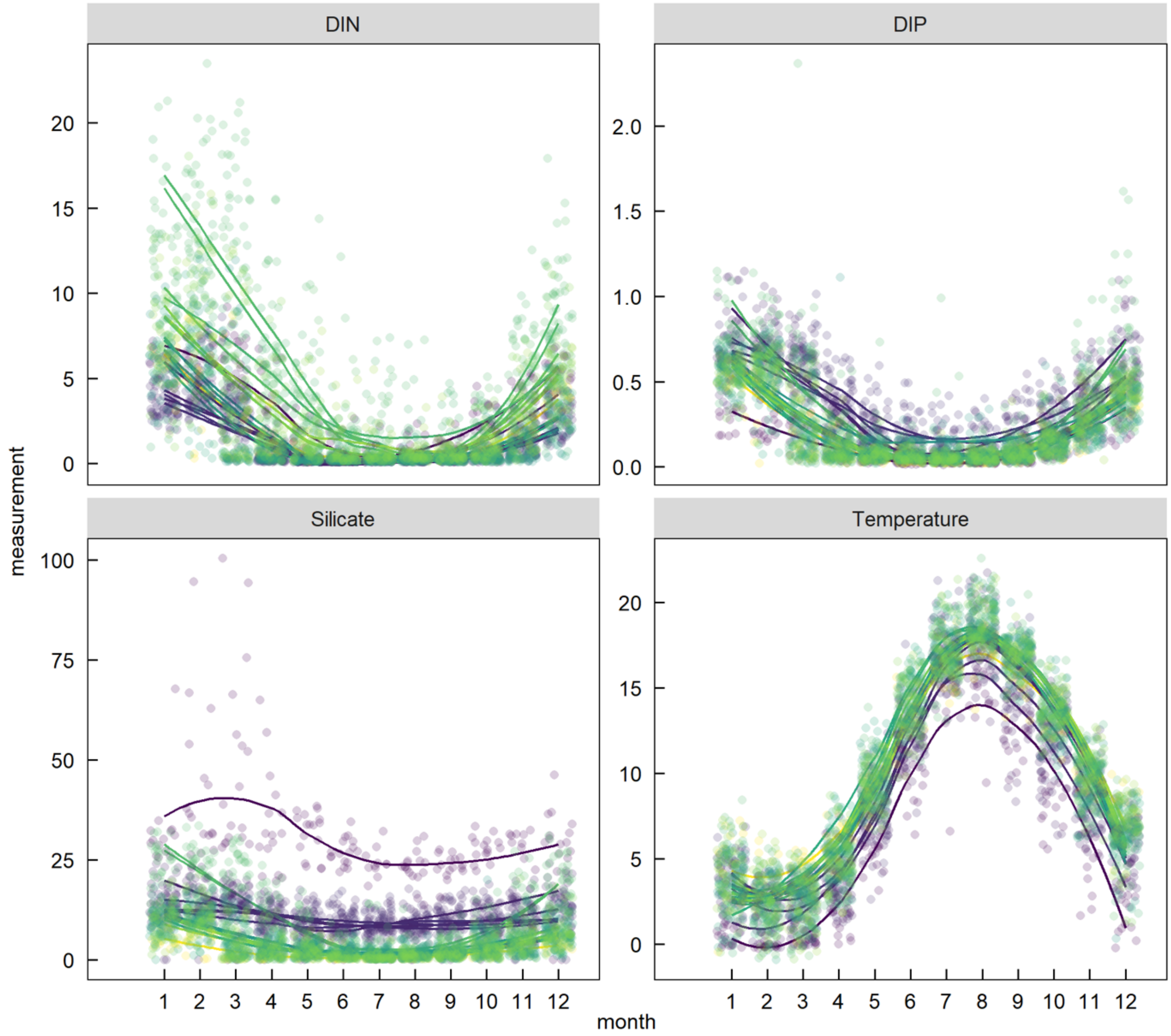

Figure S4. Seasonal environmental variation in dissolved inorganic nitrogen (DIN), dissolved inorganic phosphorus (DIP) in µM, and Sea Surface Temperature (Temperature) for stations from the Skagerrak-Kattegat (yellow circles), Baltic Sea (purples circles) and the Gulf of Bothnia (green circles). A smoothed 'lowess' curve is fit to the data each station (different line for each station). Different colours are different stations with different mean station-level salinities (colours are the same as in Fig. 1).

Relationship between diversity and cell abundance

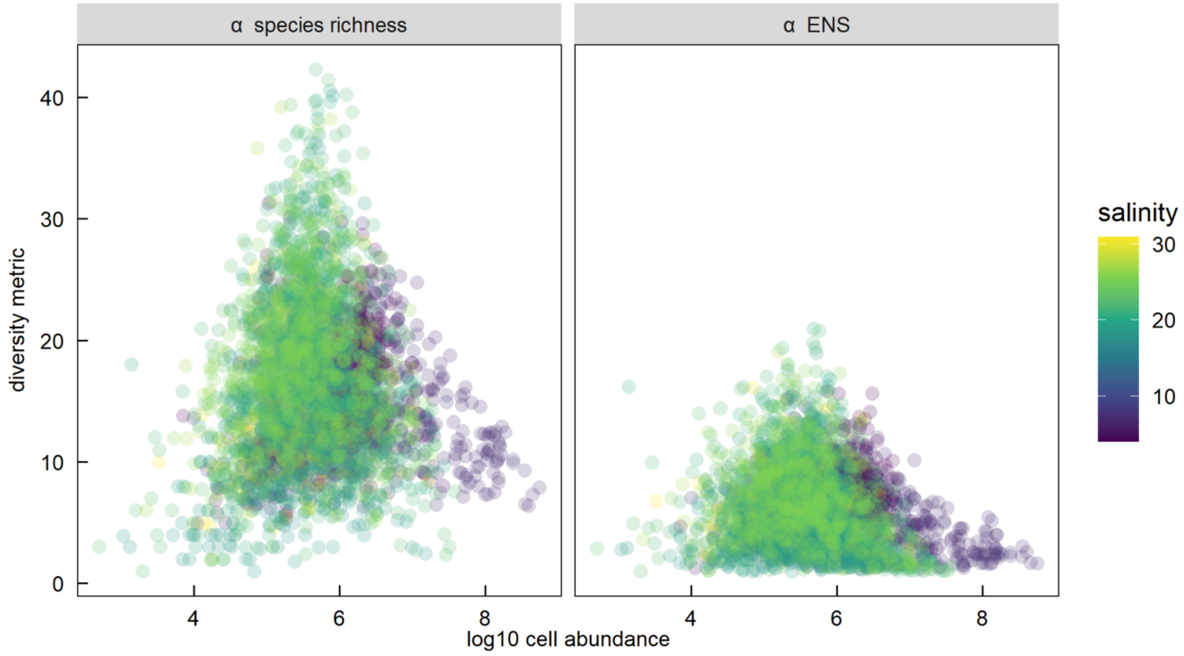

Figure S5: Relationship between log10 cell abundance with (i) α species richness (rarefied at the individual level) and (ii) α effective number of species (ENS, rarefied at the individual level). All monthly measurements of $\log_{10}$ cell abundance, α species richness and α ENS for each station are plotted. Different colours are different stations with different mean station-level salinities.

Using time-lagged environmental variables to predict seasonal variation in local-scale phytoplankton community structure

Our phytoplankton community structure and environmental variable measurements are given on a monthly timescale. Therefore, it is possible that there is a temporal mismatch. For example, phytoplankton community structure in one month may reflect the environmental conditions of the previous month. We tested this possibility by fitting the same models as reported in the main text but with ln DIN and $\sqrt[2]{\text{silicate}}$ lagged by 1 month (Table S1). Therefore, community structure measurements in one month (e.g. February 2011) were related to ln DIN and $\sqrt[2]{\text{silicate}}$ in the previous month (i.e. January 2011).

For log10 cell abundance, the best models using the time-lagged variables were worse fits to the data than the best models using the unlagged variables (marginal r^2 = [0.22 and 0.22] vs. 0.33, AIC: [6557 and 6558] vs. 6131). However, the best models using the time-lagged variables were better than the best models using unlagged variables for $\sqrt[2]{\alpha \text{ species richness}}$ (marginal r^2 = [0.05 and 0.05] vs. [0.02, 0.02, 0.02 and 0.02], AIC: [7430 and 7431] vs.[7523, 7524, 7525 and 7525]), and $\sqrt[2]{\alpha \text{ ENS}}$ (marginal r^2 = 0.22 vs. 0.33, AIC: [6189 and 6190] vs. [6286 and 6286]). Despite models using the lagged environmental variables fitting the $\sqrt[2]{\alpha \text{ species richness}}$ and $\sqrt[2]{\alpha \text{ ENS}}$ data slightly better, these models still explained little variance (<6%) and therefore our conclusions do not change.

Table S1: Best models explaining seasonal variation in three response variables: log10 cell abundance, $\sqrt[2]{\alpha \text{ species richness}}$ and $\sqrt[2]{\alpha \text{ ENS}}$. Best models were determined by AIC (ΔAIC > 2). For each response variable, all possible combinations of 1-month time-lagged ln DIN, 1-month time-lagged $\sqrt[2]{\text{silicate}}$, and the interaction between 1-month time-lagged ln DIN and mean station-level salinity were used. Mean station-level salinity was included in all models. This resulted in six models in total. When no clear model emerged, all equivalent models are reported.

model	marginal r^2	conditional r^2	AIC	AIC weight
**log10 cell abundance**				
(int.) + ln DIN + salinity + ln DIN:salinity	0.22	0.24	6557	0.71
(int.) + ln DIN + salinity + $\sqrt[2]{\text{silicate}}$ + ln DIN:salinity	0.22	0.24	6558	0.29
$\boldsymbol{\sqrt[2]{\alpha \text{ species richness}}}$				
(int.) + ln DIN + salinity + ln DIN:salinity	0.05	0.20	7430	0.64
(int.) + ln DIN + salinity + $\sqrt[2]{\text{silicate}}$ + ln DIN:salinity	0.05	0.20	7431	0.36
$\boldsymbol{\sqrt[2]{\alpha \text{ ENS}}}$				
(int.) + ln DIN + salinity + ln DIN:salinity	0.04	0.12	6189	0.56
(int.) + ln DIN + salinity + $\sqrt[2]{\text{silicate}}$ + ln DIN:salinity	0.04	0.12	6190	0.44

Methods

Comparison between species-level and genus-level diversity metrics

We tested the robustness of our species level diversity metrics by comparing it with the same metrics but calculated at the genus level. At the local monthly sample scale, observed species richness (number of species observed), rarefied species richness (number of species rarefied to the minimum observed individuals in a monthly sample) and Shannon diversity (unrarefied in this case) were strongly related the equivalent genus level metrics (Fig. S6). In all three cases, the correlation between species level and genus level metrics exceeded 0.94 (Pearson's r, Table S2). Given these strong relationships, we consider our species diversity metrics to be robust.

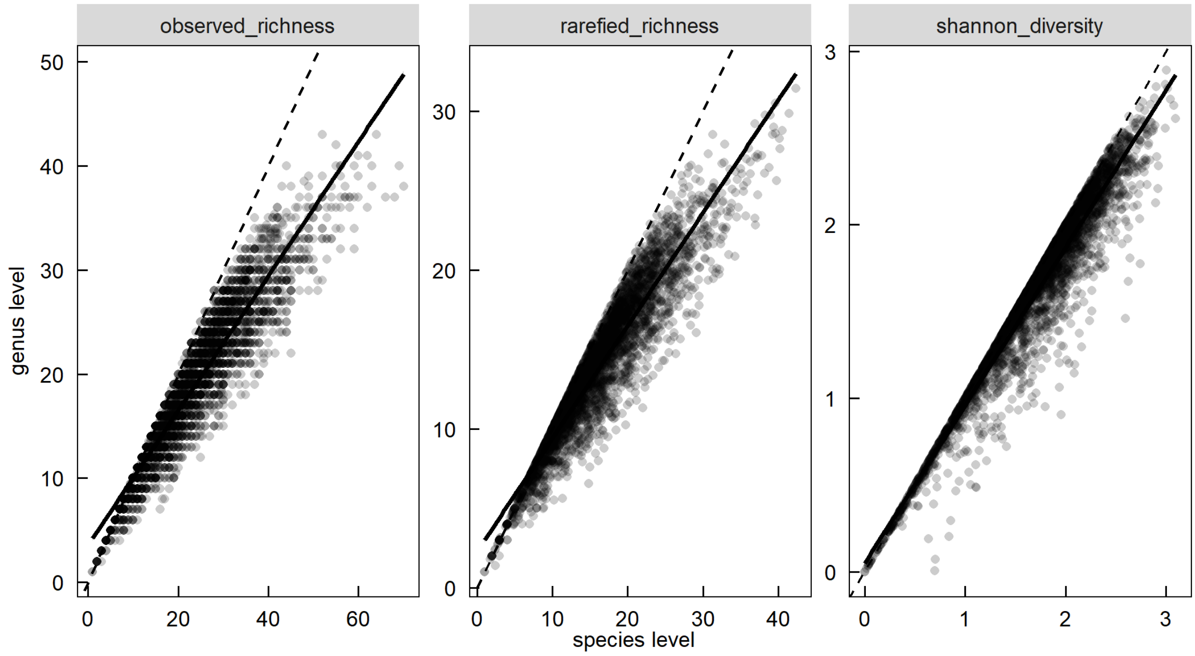

Figure S6. The relationship between species level and genus level estimates of observed richness, rarefied richness and unrarefied Shannon diversity for all monthly samples used in the analysis. Solid lines are the ordinary least squares regression line and dashed line is the 1:1 line.

Table S2. Pearson's correlation coefficients between species level and genus level estimates of observed richness, rarefied richness and unrarefied Shannon diversity for all monthly samples used in the analysis.

Diversity metric	Pearson's r	$CI_{95\%}$	t-statistic	df	P-value
Observed richness	0.94	0.93 – 0.94	165	3706	<0.001
Rarefied richness	0.94	0.93 – 0.95	170	3706	<0.001
Shannon diversity	0.97	0.967 – 0.971	240	3706	<0.001

Summary of stations used based on data availability

Location of the stations used in the study is provided in Table S3. The stations used in the study were chosen based on data availability for the addressed years and seasons, and locations are provided in Table S4.

Table S3. Station names, regions, and locations.

Region	Station	Lat. (DD°)	Lon. (DD°)
GB	Bothnian Sea (B3/B7)	63.29	19.49
BP	Arkona (BY2)	55.00	14.05
BP	Baltic Proper (B1)	58.48	17.37
BP	Baltic Proper (BY15)	57.20	20.03
BP	Baltic Proper (BY31)	58.35	18.14
BP	Baltic Proper (BY5)	55.15	15.59
SK	A17	58.17	10.31
SK	Anholt E	56.40	12.07
SK	Astol	57.55	11.36
SK	Danafjord	57.40	11.41
SK	Havstensfjord	58.19	11.46
SK	Koljofjord	58.14	11.35
SK	Kosterfjorden	58.52	11.06
SK	Laholmsbukten (L9)	56.34	12.43
SK	Falkenberg (N14)	56.56	12.12
SK	Ost Nidingen (N7)	57.18	11.59
SK	S5	56.19	12.39
SK	Slaggo	58.16	11.26
SK	Stretudden	58.21	11.24

Table S4: Overview of the number of the data completeness for the three groups of analyses performed (see Materials and methods). For each station and each group of analyses, the range in years, number of monthly samples (n) and a completeness measure are shown. Completeness is defined as the number of monthly samples divided by the total number of possible monthly in the year range for that station.

	analysis 1			analysis 2			analysis 3		
station name	year range	n	completeness	year range	n	completeness	year range	n	completeness
A17	2001-2017	173	0.85	2001-2017	154	0.75	2001-2017	173	0.85
Anholt E	2000-2017	194	0.90	2000-2017	182	0.84	2000-2017	182	0.84
Arkona BY2	1999-2017	200	0.88	1999-2017	175	0.77	1999-2017	200	0.88
Astol	1999-2017	205	0.90	1999-2017	193	0.85	2010-2017	86	0.90
Baltic Proper B1	1999-2017	213	0.93	1999-2017	209	0.92	1999-2017	213	0.93
Baltic_Proper BY15	1999-2017	190	0.83	1999-2017	166	0.73	1999-2017	190	0.83
Baltic_Proper BY31	1999-2017	209	0.92	1999-2017	193	0.85	1999-2017	209	0.92
Baltic_Proper BY5	1999-2017	192	0.84	1999-2017	170	0.75	1999-2017	192	0.84
Bothnian Sea B3 B7	1999-2017	183	0.80	1999-2017	161	0.71	1999-2017	183	0.80
Danafjord	1999-2017	211	0.93	1999-2017	195	0.86	2009-2017	92	0.85
Havstensfjord	1999-2017	209	0.92	1999-2017	193	0.85	2009-2017	92	0.85
Koljofjord	1999-2017	211	0.93	1999-2017	197	0.86	2009-2017	93	0.86
Kosterfjorden	1999-2017	201	0.88	1999-2017	179	0.79	2001-2017	84	0.41
L9 Laholmsbukten	2000-2017	197	0.91	2000-2017	180	0.83	2004-2017	148	0.88
N14 Falkenberg	2000-2017	133	0.62	2000-2017	121	0.56	2008-2017	109	0.91
N7 Ost Nidingen	2000-2017	190	0.88	2000-2017	163	0.75	2005-2017	137	0.88
S5	1999-2017	184	0.81	1999-2017	160	0.70	NA	NA	NA
Slaggo	2000-2017	213	0.99	2000-2017	209	0.97	2000-2017	213	0.99
Stretudden	2000-2017	200	0.93	2000-2017	186	0.86	2009-2017	93	0.86
